# Personalized Prediction of Clozapine Treatment Response Using Therapeutic Drug Monitoring Data in Japanese Patients with Treatment-Resistant Schizophrenia

**DOI:** 10.3390/jcm14217892

**Published:** 2025-11-06

**Authors:** Tatsuo Nakahara, Yukiko Harada, Naho Nakayama, Kijiro Hashimoto, Naoya Kida, Toshiaki Onitsuka, Hiroo Noda, Kenji Murasugi, Yoshihiro Takimoto, Wataru Omori, Tsuruhei Sukegawa, Jun Shiraishi, Kouji Tanaka, Hitoshi Maesato, Takefumi Ueno

**Affiliations:** 1 National Hospital Organization, Hizen Psychiatric Center, Saga 842-0192, Japan; nakahara@ruby.plala.or.jp (T.N.); yukiko.harada401@gmail.com (Y.H.); naho.nakayama.h259@gmail.com (N.N.); kijiro1@icloud.com (K.H.); 2 National Hospital Organization, Ryukyu Hospital, Okinawa 904-1201, Japan; naoyakida7@gmail.com (N.K.); jintan@chive.ocn.ne.jp (H.M.); 3 National Hospital Organization, Sakakibara Hospital, Mie 514-1292, Japan; onitsuka.toshiaki.vc@mail.hosp.go.jp; 4 National Hospital Organization, Saigata Medical Center, Niigata 949-3193, Japan; noda.hiroo.kf@mail.hosp.go.jp; 5 National Hospital Organization, Komoro Kogen Hospital, Nagano 384-8540, Japan; murasugi.kenji.qu@mail.hosp.go.jp; 6 National Hospital Organization, Yamato Psychiatric Center, Nara 639-1042, Japan; takimoto.yoshihiro.nm@mail.hosp.go.jp; 7 National Hospital Organization, Department of Psychiatry, Kure Medical Center and Chugoku Cancer Center, Hiroshima 737-0023, Japan; watsu56@yahoo.co.jp; 8 National Hospital Organization, Tottori Medical Center, Tottori 689-0203, Japan; sukegawa@mmwc.or.jp; 9 National Hospital Organization, Hokuriku Hospital, Toyama 939-1893, Japan; shiraishijun@gmail.com; 10 National Hospital Organization, Kikuchi National Hospital, Kumamoto 861-1116, Japan; tanaka.kouji.ed@mail.hosp.go.jp; 11 National Hospital Organization, Kurihama Medical and Addiction Center, Kanagawa 239-0841, Japan

**Keywords:** clozapine, norclozapine, clozapine N-oxide, therapeutic drug monitoring (TDM), random forest (RF), personalized medicine, treatment-resistant schizophrenia (TRS)

## Abstract

**Background**: Clozapine is the only antipsychotic medication proven effective in patients with treatment-resistant schizophrenia (TRS). However, many patients have serum concentrations outside the recommended therapeutic window, and clozapine exhibits substantial interindividual variability. This study aimed to (1) examine clozapine dosage and blood concentrations in patients with TRS; (2) investigate the effects of sex and age on dosage and blood concentrations; (3) assess clinical response to clozapine treatment; and (4) develop a random forest (RF) model to predict therapeutic response using clinical and therapeutic drug monitoring (TDM) data. **Methods:** Dried blood spots were used to measure concentrations of clozapine, norclozapine, and clozapine N-oxide. Clinical symptoms were assessed using the Brief Psychiatric Rating Scale (BPRS). The RF algorithm was applied to analyze the relationships between biochemical and demographic factors and clinical response to clozapine. **Results:** A total of 754 blood samples from 167 patients were analyzed. Men received higher doses than women, and glucose levels were elevated in both sexes. The area under the curve (AUC) of the receiver operating characteristic (ROC) curve was 0.986 for the training set and 0.852 for the testing set. Accuracy, precision, recall, and F1-score (training/testing) were 0.938/0.786, 0.936/0.736, 0.934/0.780, and 0.935/0.757, respectively. The SHapley Additive exPlanations (SHAP) analysis indicated that baseline BPRS score, treatment duration, age, and clozapine concentration were important variables contributing to the output of the model. **Conclusions:** Our model achieved satisfactory predictive performance for clinical response and provides valuable insights into personalized prediction of clozapine efficacy.

## 1. Introduction

Schizophrenia is a severe psychiatric disorder characterized by cognitive dysfunction, and positive and negative symptoms. Approximately 20–30% of patients with schizophrenia develop treatment-resistant schizophrenia (TRS), which is defined as an insufficient response to at least two trials of different antipsychotics administered at adequate dose and duration. Clozapine is the only antipsychotic with established efficacy for TRS and is recommended by treatment guidelines as the most effective option for these patients. However, its use requires careful monitoring because of potential adverse effects such as agranulocytosis and metabolic disturbances. Clozapine is the most effective treatment for TRS; however, its efficacy varies considerably among individuals. Since serum clozapine concentrations are related to clinical response, therapeutic drug monitoring (TDM)—which involves measuring drug levels in patients—is valuable for optimizing dosage and minimizing adverse effects [1]. The AGNP Consensus Guidelines recommend maintaining plasma clozapine concentrations within the range of 350–600 ng/mL, which is widely accepted as the therapeutic reference range associated with optimal efficacy and safety [2,3]. Although this reference range can help estimate the clinical response, many patients fall outside the therapeutic window. Approximately one-third (31–33%) of patients achieve therapeutic clozapine levels of 350–600 ng/mL, whereas 26–36% have levels below 350 ng/mL and 31–45% exceed 600 ng/mL [4,5]. The potential barriers to achieving optimal levels may be factors influencing clozapine concentration including dose, sex, age, body weight, smoking and comedication, and insufficient monitoring.

Several studies have reported a correlation between clozapine plasma concentration and clinical response [2,3,4]; however, this correlation has not been consistently observed [6,7,8,9]; the reported *p*-values for the correlation between clozapine concentration and clinical response were 0.33 [6], not reported in [7], 0.98 [8], and 0.62 [9]. Similarly, norclozapine levels and the clozapine-to-norclozapine concentration (clozapine/norclozapine) ratio have been reported to be associated with clinical outcomes [6,10]. Furthermore, the demographic characteristics or clinical functions such as sex [11], dose [10,12], clozapine concentration-to-dose (clozapine/dose) ratio [10], treatment duration [6,10,12], severity of illness at baseline [13,14,15,16,17] somatic medication [11], social function, and shorter illness duration [7] have been shown to affected the clinical efficacy of clozapine. These findings suggest that individualized dosing strategies may be more appropriate than applying a uniform dosing regimen across all patients [8]. Therefore, predicting clinical response should be personalized to account for the interindividual variations in the efficacy of clozapine. The present study aimed to predict the relationship between clozapine and norclozapine blood concentrations and clinical outcomes, thereby supporting a more personalized and clinically effective strategy for TDM.

Machine learning (ML) has been applied to predict antipsychotic treatment outcomes in patients with psychiatric disorders [18]. Several ML approaches have been used to predict responses to clozapine treatment [7,8,12]. In this study, we applied a ML model using TDM data to predict the therapeutic response to clozapine and to determine the optimal treatment strategy in individual patients with TRS. The recent review for the efficacy of clozapine versus second-generation antipsychotics using individual patient data suggested that future studies should include participants with strictly defined TRS, monitor both clinical response and plasma concentrations, and explore inter-individual variability in response to clozapine [19]. Our study emphasizes the importance of personalized prediction of response to clozapine for optimizing dosing in patients with TRS.

Therefore, the present study has the following aims:

(1) Examine clozapine dosage and blood concentrations in patients with treatment-resistant schizophrenia (TRS);
(2) Investigate the effects of sex and age on dosage and blood concentrations;
(3) Evaluate blood profiles and neutrophil counts;
(4) Assess clinical response to clozapine treatment;
(5) Develop a random forest model to predict therapeutic response using clinical and TDM data.

## 2. Materials and Methods

### 2.1. Design and Participant

For patients with TRS, baseline assessments, including patient background, pre-clozapine medication status, and clinical symptom evaluations (BPRS, CGI-C, and GAF), were performed prior to treatment initiation. Clozapine concentrations were measured at weeks 4, 12, 24, and 48 after the start of treatment. At each time point, dosage of clozapine, treatment continuation status, and clinical evaluations were assessed.

This study was conducted from January 2017 to June 2025. Patients were enrolled from 11 National Hospital Organization institutions in Japan: Hizen Psychiatric Center; Ryukyu Hospital; Sakakibara Hospital; Saigata Medical Center; Komoro kogen Hospital; Yamato Psychiatric Center; Kure Medical Center; Tottori Medical Center; Hokuriku Hospital; Kikuchi National Hospital; and Kurihama Medical and Addiction Center.

All patients were diagnosed according to the Diagnostic and Statistical Manual of Mental Disorders, fifth edition (DSM-5) [20] criteria, meeting the criteria for treatment-resistant schizophrenia, characterized by an inadequate response to two different antipsychotics [21]. Information collected included diagnosis, sampling date, clozapine dose (mg/day), clozapine treatment duration, age, sex, body weight, height, body mass index (BMI), fasting glucose, hemoglobin A1c (HbA1c), total cholesterol (TC), triglycerides (TG), and high-density lipoprotein (HDL) cholesterol levels. White blood cell (WBC), neutrophil (NEUT), alanine aminotransferase (ALT; glutamic pyruvate transaminase), aspartate aminotransferase (AST; glutamic oxaloacetic transaminase), and γ-glutamyl transpeptidase (γ-GTP) were monitored at blood sampling. In this study, treatment was discontinued in some cases due to adverse events or side effects including neutropenia, leukopenia, increased serum creatinine levels, paralytic ileus, seizure, acute liver failure, abnormal test results, and medication refusal. Data from these cases were obtained before disease onset. Therefore, disease effects were considered negligible.

This study was approved by the Ethics Committee of Hizen Psychiatric Center, and written informed consent was obtained from all participants.

### 2.2. Measurement of Blood Levels

Dried blood spots (DBSs) of whole blood were used to measure the concentrations of clozapine, norclozapine (N-desmethylclozapine), and clozapine N-oxide. Samples were mailed from the participating hospitals to Hizen Psychiatric Center, where DBS samples were analyzed as previously described [22]. In this method, volumetric absorptive paper discs (VAPDs, 6 mm in diameter) were used; samples were pretreated using liquid–liquid extraction with ethyl acetate. Using another method, VAPDm6, with a paper disc diameter of 6 mm (sampling blood volume: 25.0 μL), was used instead of the VAPDmini disc (3 mm diameter, 4.8 μL sampling volume) [22]. In this approach, target analytes were directly extracted using a methanol solution of phosphate buffer without liquid–liquid extraction. In brief, the measurements were performed in duplicate. The VAPDm6 disc was removed from the sheet and placed into a 96-well microplate; 250 μL of a 50% methanol solution in 50 mM phosphate buffer (pH 2.1) was added. The plate was then sealed, and the sample was sonicated for 30 min at 30 °C and centrifuged for 4 min. Concentrations were determined using high-performance liquid chromatography (HPLC), as previously reported [22].

### 2.3. Clinical Assessment

Clinical symptoms were assessed using the BPRS by experienced clinicians within several days of blood sampling. The BPRS is an 18- or 24-item rating scale that includes six subscales: affect, positive symptoms, negative symptoms, activation, resistance, and disorganization. Scores obtained using the 18-item version were converted to the corresponding 24-item scores, allowing comparisons of both the total and subscale scores between versions.

### 2.4. Statistical Analysis

Our data did not follow a normal distribution; therefore, the Mann–Whitney U test was used for between-group comparisons. Analyses were performed using the JMP 18.1.1 software package (SAS Institute, Cary, NC, USA).

### 2.5. Machine Learning

The model was implemented in a Python 3.11 environment using the Random Forest Regressor from the scikit-learn library for training and feature importance analysis [23,24]. SHAP values were computed with the TreeExplainer function from the shap package, applied to the trained Random Forest Regressor model [25]. ROC curves, accuracy, precision, and recall were calculated by defining clinical response as a ≥20% reduction in BPRS total scores. For predicting clinical efficacy, the following were selected as explanatory variables: age, sex, dosage, treatment duration, baseline BPRS total scores, concentrations of clozapine, norclozapine, clozapine-N-oxide, clozapine/norclozapine ratio, and clozapine/dose ratio. The BPRS reduction rate (%) or total BPRS score after clozapine treatment was used as the objective variable.

The performance of the model was evaluated using AUC, accuracy, precision, recall, and F1-score. The model was trained on 75% and tested on 25% of the data, and optimized using four-fold cross-validation. SHAP, a theoretically reliable and intuitive method for explaining ML models’ outcomes, was used to visualize the importance of each variable in the RF model. The SHAP value quantifies the contribution (importance) of that variable to the prediction made by the model, and the direction (positive or negative) and magnitude of its influence.

## 3. Results

### 3.1. Clozapine Dosage and Concentration in Blood

**Figure 1:**
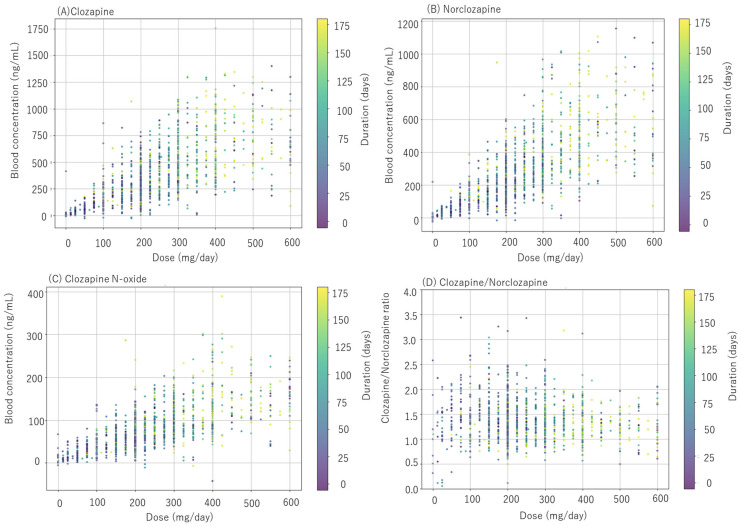
Relationship between the daily clozapine dose and blood concentrations of (**A**) clozapine, (**B**) norclozapine, (**C**) clozapine N-oxide, and (**D**) the clozapine/norclozapine ratio. The color of each point indicates treatment duration.

A total of 754 blood samples were collected from 167 patients in this study. The distribution of samples per patient was 1 to 3 samples (28 patients); 4 or 5 samples (98 patients); 6 to 11 samples (41 patients), with an average of 4.53 ± 1.53 (mean ± standard deviation [SD]) samples per patient. Treatment durations were 4–8 weeks (10 patients), 9–24 weeks (51 patients), 25–48 weeks (65 patients), and 49–72 weeks (41 patients) with an overall average of 38.0 ± 17.0 weeks. The dosage and blood concentrations at all measurement points and at the final treatment are presented in Table 1. The averages among 167 patients at the endpoint were as follows: dose (324 ± 122 mg/day), clozapine (526 ± 261 ng/mL), norclozapine (399 ± 200 ng/mL), clozapine N-oxide (108 ± 52 ng/mL), clozapine/norclozapine ratio (1.35 ± 0.33) and clozapine/dose ratios (1.70 ± 0.80). Clozapine, norclozapine, and clozapine N-oxide concentrations, as well as the clozapine/norclozapine ratio, were plotted against dose and treatment duration (Figure 1). Clozapine concentration increased with both higher doses and longer treatment durations. Clozapine concentrations varied 25-fold (mean ± SD (*n*), range, ng/mL) at 200 mg/day (326 ± 153 (144), 32–795 ng/mL), 15-fold at 300 mg/day (535 ± 251 (106), 10–1515 ng/mL), 5.6-fold at 400 mg/day (665 ± 254 (69), 237–1331 ng/mL), and 6.0-fold at 600 mg/day (709 ± 324 (69), 228–1372 ng/mL).

### 3.2. Sex and Age Effects on Dosage and Blood Concentrations

A total of 100 men (aged 40.5 ± 11.3 years, range 18–68 years) and 67 women (aged 41.1 ± 11.8 years, range 18–72 years) were included in the study. Men received significantly higher clozapine doses than women both before the endpoint (288 ± 130 vs. 252 ± 115 mg/day, *p* < 0.001) and at the endpoint (344 ± 125 vs. 295 ± 114 mg/day, *p* = 0.011) (Table 2). However, no significant sex differences were observed in clozapine, norclozapine, or clozapine N-oxide levels concentrations, or the clozapine/norclozapine ratio, either before or at the endpoint.

Patients were further divided into two age groups: 83 patients aged < 40 years (31.2 ± 5.3 years, range 18–39 years) and 84 patients aged ≥ 40 years (50.1 ± 7.5 years, range 40–72 years). No significant differences were observed between the two groups in clozapine dose, clozapine, norclozapine, or clozapine N-oxide concentrations, or the clozapine/norclozapine ratio (Table 3).

### 3.3. Blood Profiles and Cell Counts

In Table 4, blood parameters and cell counts for men and women are presented separately. Differences between baseline and endpoint values were analyzed using the Mann–Whitney U test. Glucose levels increased significantly following clozapine treatment in both sexes, with *p*-values of 0.018 for men and 0.023 for women. HDL cholesterol levels increased significantly in men (*p* = 0.025), but not in women (*p* = 0.440). No significant changes were observed in body weight, BMI, HbA1c, total cholesterol (TC), HDL cholesterol, or triglyceride (TG) levels between baseline and endpoint. Similarly, no significant changes were found in cell counts (WBC, NEUT) or liver enzyme levels (ALT, AST, and γ-GTP) before and after clozapine treatment.

### 3.4. Clinical Response

**Figure 2:**
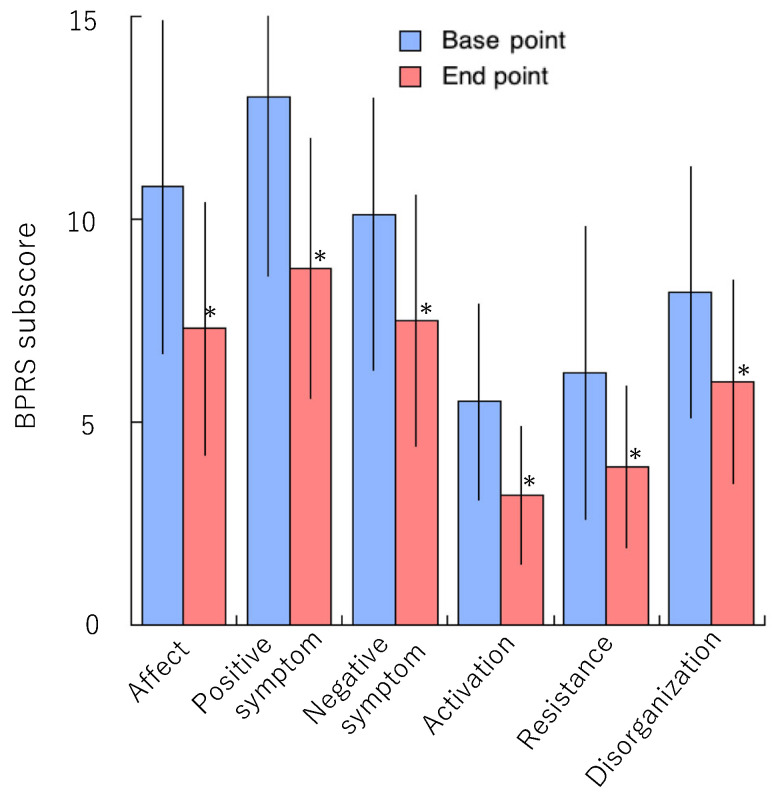
BPRS subscale scores at baseline (before treatment) and at endpoint (after treatment) with clozapine. Mann–Whitney U test: * *p* < 0.0001 vs. baseline.

The BPRS total scores were significantly reduced from baseline to endpoint (71.8 ± 19.9 vs. 48.9 ± 16.7, *n* = 167; *p* < 0.0001), as were all BPRS subscale scores (Figure 2). At baseline, subscale scores for affect, and positive and negative symptoms were high, whereas activation and resistance subscale scores were low.

### 3.5. Random Forest Model Performance for Predicting Clozapine Response

**Figure 3:**
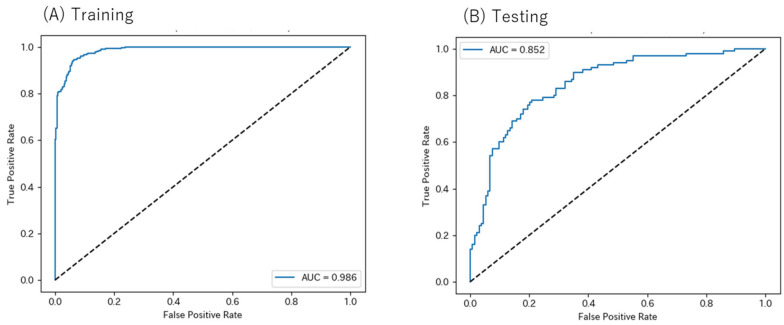
ROC curves for predicting clinical response to clozapine in the training dataset (**A**) and the testing dataset (**B**).

**Figure 4:**
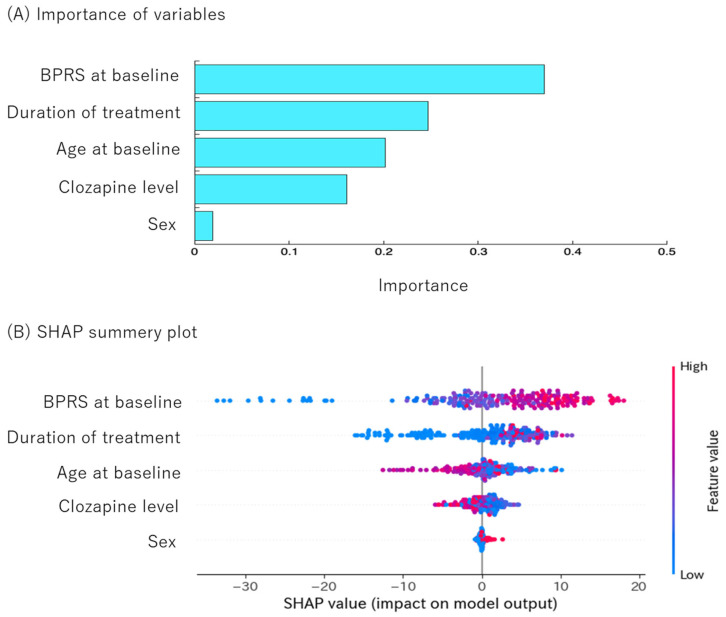
Variable importance (**A**) and individual SHAP values (**B**).

ROC curves illustrating the relationship between explanatory variables and clinical response are shown in Figure 3. AUC was 0.986 for the training set and 0.852 for the testing set. The accuracy, precision, recall, and F1-score were 0.938, 0.936, 0.934, and 0.935 for the training set and 0.786, 0.736, 0.780, and 0.757 for the testing set, respectively. In Figure 4, the importance of each variable (panel A) and the SHAP summary plots (panel B) are shown for the five most influential variables in predicting the clinical response to clozapine. The most important variable was baseline BPRS total scores, followed by treatment duration, age, clozapine concentration, and sex. The bar plots indicate the contribution of each variable to the prediction made by the random forest model, with higher mean absolute SHAP values reflecting a greater contribution to model output. The SHAP summary plots visualize how low (blue), intermediate, and high (red) values of each variable are associated with the probability of clinical improvement.

Increases in baseline BPRS total score and treatment duration were associated with clinical improvement, whereas younger age was associated with a higher probability of response. Clozapine concentration was positively associated with clinical response at lower concentrations but negatively associated at higher concentrations. Daily dose, norclozapine and clozapine N-oxide concentrations, the clozapine/norclozapine ratio, and the clozapine/dose ratio were not significantly associated with clinical response.

**Figure 5:**
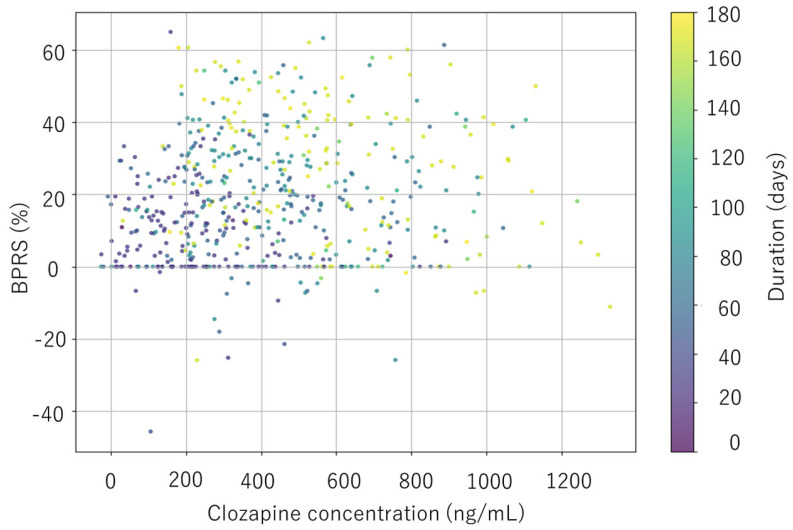
Relationship between clozapine concentrations and reduction in BPRS total scores. The color of each point indicates treatment duration.

Figure 5 shows the relationship between clozapine concentrations and the reduction in BPRS total scores. The color of each point indicates treatment duration. Clozapine concentration was positively associated with clinical response at lower concentrations and negatively associated at higher concentrations, consistent with the SHAP findings.

## 4. Discussion

We developed a personalized prediction model for clozapine response using TDM data from Japanese patients with TRS. Our analysis indicated that baseline BPRS scores and treatment duration were the most important variables for predicting clinical outcomes. Age and clozapine concentration also consistently contributed to the prediction of clinical response. In contrast, we did not find consistent associations between clozapine dose, the clozapine/dose ratio, or the clozapine/norclozapine ratio and clinical outcome.

In the present study, only 63 patients (37.5%) had blood clozapine levels within the proposed therapeutic range (350–600 ng/mL) [2], whereas 48 patients (28.5%) had levels below the lower limit (<350 ng/mL), and 57 patients (33.9%) had levels exceeding the upper limit (600–1200 ng/mL) at the final assessment point. In contrast, 133 patients (79.1%) had blood norclozapine levels within the therapeutic range (100–600 ng/mL) [26]. The percentage of patients with clozapine levels within the proposed therapeutic range (33.9%) was consistent with previous reports, which showed rates of 22% [26], 30% [27], 31% [5], 32% [28], 33% [29], and 37% [30]. Therefore, establishing a clear association between clozapine concentration and clinical response is essential to ensure that clozapine levels are maintained within the effective therapeutic range [10].

The average whole-blood concentrations of clozapine and norclozapine before the endpoint were 457 ng/mL and 340 ng/mL, respectively. These values were consistent with previous findings, accounting for the known differences in clozapine and norclozapine concentrations between whole blood and serum. Previous studies have shown that serum concentrations of clozapine and norclozapine are approximately 40% higher and 17% lower, respectively, than their corresponding whole-blood concentrations [31]. This difference is likely due to the binding of clozapine derivatives to α_1_-acid glycoprotein [32]. Similarly, we confirmed the relationship between whole-blood (*x*) and serum (*y*) levels in our data: clozapine, *y* = 1.43*x* − 51.1, *n* = 31, *R*^2^ = 0.8926; norclozapine, *y* = 0.92*x* − 14.4, *n* = 31, *R*^2^ = 0.9056. Using these equations, the average whole-blood concentrations corresponded to estimated serum concentrations of 602 ng/mL for clozapine and 298 ng/mL for norclozapine. These results were consistent with previous reports of serum concentrations of clozapine (533 ng/mL) and norclozapine (271 ng/mL) [32].

Clozapine dosages in male patients were consistently higher than those in female patient timepoints, including the endpoint, which is consistent with other studies [27,33,34]. However, there was no significant difference in clozapine or its metabolite concentrations between male and female patients. The difference in dosage did not affect the blood concentrations. The sex effects on clozapine levels may be explained by lower cytochrome P4501A2 (CYP1A2) activity in women and physiological factors such as higher body fat percentage and slower renal clearance [29]. However, we did not find a sex-related difference in the clozapine/norclozapine concentration ratio, suggesting no significant variation in the metabolism of these compounds between men and women. When considering sex effects on clozapine and norclozapine levels, the variation in smoking habits between men and women should also be taken into account [35,36,37]. There was no significant difference in the concentrations of clozapine, norclozapine, and clozapine N-oxide between patients aged ≥40 years and those aged <40 years. These findings are consistent with some previous studies [29,38], though not all [39]. It is suggested that with increasing age, renal and hepatic clearance may decline, while the volume of distribution may increase [27].

Glucose levels increased in both sexes, whereas HDL cholesterol levels increased only in men. The elevation in glucose levels may be associated with impaired insulin secretion, insulin resistance, or disruption of glucose homeostasis [40]. These findings suggest clozapine-induced metabolic dysregulation, consistent with previous studies reporting a high prevalence of metabolic side effects associated with clozapine treatment [41]. Some studies have also reported that elevated serum lipid levels—such as total cholesterol (TC), triglycerides (TG), and HDL cholesterol—are associated with clinical improvement in patients treated with clozapine [42]. No reduction in neutrophil (NEUT) counts was observed following clozapine treatment, suggesting that clozapine-induced hematotoxicity may not result from the direct toxicity of clozapine or its metabolite, norclozapine, on blood cells [43]. However, potential confounding factors such as physical comorbidities, smoking, diet, physical activity, and the co-prescription of psychiatric or physical medications should be considered when interpreting these results [44].

Machine learning methods have been used to predict the efficacy of psychopharmacologic treatments based on fMRI and EEG data [18], and to support TDM and model-informed precision dosing (MIPD) [45]. In contrast, we used clinical factors and TDM data to predict pharmacological response. Baseline BPRS scores were the most important predictor in our model; higher baseline scores were associated with greater reductions in follow-up BPRS scores, suggesting that initial symptom severity may be associated with enhanced responsiveness to clozapine. This finding implies that patients with greater baseline symptom severity might derive particular benefit from clozapine treatment. The assessment of initial clinical severity is important for personalized therapeutic strategies and monitoring treatment outcomes. The association between severity at baseline and clozapine efficacy at follow-up is in line with previous studies [13,46,47]. Moreover, a recent study demonstrated that baseline BPRS as a predictor significantly increased logistic regression model (performance and concordance up to 92%) for predicting clinical improvement using resting-state fMRI, and the antipsychotic dosage was not associated with the clinical outcomes [48].

Treatment duration was also a key predictor of clozapine response. Scatter plots showing the relationship among clozapine concentration BPRS reduction (%) and treatment duration indicated that shorter duration was associated with clinical response (BPRS decrease ≥ 20%) at clozapine levels below 200 ng/mL, but long-term treatment predicted better clinical outcome at 200–600 ng/mL (Figure 5). Several studies showed that clozapine treatment duration was significantly related to clinical response [6,10,12]. Age was a predictor in our study, as indicated in SHAP plots (Figure 4). This finding is consistent with some previous studies [39,49,50], though not all [13,29,37,38]. It is suggested that with increasing age, renal and hepatic clearance may decline while the volume of distribution may increase [27]. Clozapine concentration was a predictor of treatment response; lower plasma levels were associated with improved clinical outcomes, while higher levels were linked to reduced efficacy, as shown in Figure 4. These findings suggest that although lower concentrations may enhance clinical response, higher concentrations might lead to poorer outcomes. One possible explanation is that poor responders were administered higher doses, potentially resulting in adverse side effects that counteracted therapeutic benefits. The correlation between clozapine level and clinical response has been reported [3,10,11,51], though this relationship has not always been consistent [6,7,8,9]. We did not find the association of dose, norclozapine concentration, and clozapine/norclozapine ratio with clinical outcome. Previous studies have suggested that dose [3,9,51], norclozapine levels [6,7,9], clozapine/dose ratio [3,11,51], and clozapine/norclozapine ratio [8,9] do not improve the association between clozapine levels and clinical response; however, these findings have not been consistently confirmed [6,10,12]. A recent review has hypothesized a negative correlation between clozapine/norclozapine level ratio and cognitive functioning [52]. In summary, the severity of syndromes at baseline, treatment duration, age at starting treatment, and clozapine concentration are suggested to be important for personalized prediction of clozapine outcomes.

In this study, dried blood spot samples were used. It is anticipated that home-care patients could perform self-blood sampling, send the samples to the hospitals via regular mail, and based on the analysis results, healthcare providers could predict the therapeutic effect in a personalized and more detailed manner [53].

The introduction of clozapine in Japan remains extremely limited [54], compared with other countries [55]. To address the concerns of both patients and healthcare providers about its use, we investigated the potential role of TDM. Due to the significant individual variability in blood concentrations, which could not be predicted solely based on dosage, we employed the RF model to improve prediction accuracy using TDM data. Predictions for clinical responses and prognosis were feasible using this model. Our findings suggested that clozapine TDM, combined with growing clinical experience, could aid in determining optimal treatments in individual patients. This approach might help overcome the barriers to clozapine adoption.

The present study has some limitations. First, the number of patients and samples was not very large. Due to missing values in certain measurements, we had to evaluate a smaller number of participants for specific items, and caution is required when interpreting the results. Second, the RF model showed slightly higher performance in the single-center trial (conducted at the hospital with the largest sample size) compared with the multi-center trials (across all hospitals). This discrepancy may stem from differences in clinical assessments among facilities. In future studies, heterogeneity in clinical symptom evaluations should be carefully addressed prior to study initiation. Third, we did not deploy any sort of analysis of agreement between those who assessed the severity of symptoms. There could be a bias that might be potentially relevant to disclose. Fourth, the duration of illness was not included as a variable in the RF analysis due to missing data. Finally, this study could not estimate the effect of smoking on clinical outcomes because smoking history was unavailable.

## 5. Conclusions

We observed that men received higher doses of clozapine than women, and glucose levels increased in both sexes. Using therapeutic drug monitoring (TDM) data from Japanese patients with treatment-resistant schizophrenia (TRS), we developed a random forest (RF) model to predict the therapeutic efficacy of clozapine. Symptom severity, duration of treatment, age, and clozapine blood levels emerged as important predictors of treatment outcomes. These findings highlight sex-related and metabolic differences associated with clozapine use. The proposed prediction model may support personalized treatment by estimating therapeutic response and helping determine optimal therapeutic strategies for individual patients with TRS.

## Figures and Tables

**Table 1:** Demographic characteristics of patients and clozapine dosage, clozapine, norclozapine, and clozapine N-oxide concentrations before endpoint and at endpoint.

	Mean	SD	Range
Patients, sample number, and treatment duration (*n* = 167)			
Age (y)	40.6	11.4	18–72
Number of samples/patients	4.53	1.53	1–11
Treatment duration (w)	38.4	17.0	4–72
Dose and concentrations before endpoint (*n* = 587)			
Dose (mg/day)	275	126	13–600
Clozapine (ng/mL)	457	282	38–1372
Norclozapine (ng/mL)	340	221	36–1319
Clozapine N-oxide (ng/mL)	90	50	10–390
Clozapine/norclozapine ratio	1.39	0.51	0.78–7.57
Clozapine/dose ratio	1.68	0.79	0.37–5.48
Dose and concentrations at endpoint (*n* = 167)			
Dose (mg/day)	324	122	75–600
Clozapine (ng/mL)	526	261	106–1515
Norclozapine (ng/mL)	399	200	101–1131
Clozapine N-oxide	108	52	33–295
Clozapine/norclozapine ratio	1.35	0.33	0.79–2.48
Clozapine/dose ratio	1.70	0.80	0.10–5.05

**Table 2:** Sex differences in clozapine dose and blood concentrations of clozapine, norclozapine, and clozapine N-oxide, as well as clozapine/norclozapine and clozapine/dose ratios.

Sex	Men (*n* = 100)			Women (*n* = 67)			*p*
	Mean	SD	Range	Mean	SD	Range	
Dose and concentrations before endpoint (*n* = 373/214)							
Dose (mg/day)	288	130	13–600	252	115	50–600	<0.001
Clozapine (ng/mL)	463	271	45–1372	446	300	38–1331	0.205
Norclozapine (ng/mL)	349	216	38–1100	326	229	33–1319	0.094
Clozapine N-oxide (ng/mL)	90	45	10–242	89	58	25–390	0.197
Clozapine/norclozapine ratio	1.37	0.48	0.80–3.43	1.42	0.56	0.78–7.57	0.215
Clozapine/dose ratio	1.63	0.75	0.37–5.04	1.76	0.85	0.37–5.48	0.063
Dose and concentrations at end points (*n* = 100/67)							
Dose (mg/day)	344	125	100–600	295	114	75–525	0.011
Clozapine (ng/mL)	525	228	161–1119	528	307	106–1515	0.752
Norclozapine (ng/mL)	398	177	129–913	401	231	101–1131	0.753
Clozapine N-oxide (ng/mL)	110	46	39–263	106	60	33–295	0.255
Clozapine/norclozapine ratio	1.36	0.33	0.83–2.48	1.33	0.32	0.79–2.41	0.795
Clozapine/dose ratio	1.61	0.65	0.52–3.46	1.82	0.97	0.10–5.05	0.199

*p*-value: Mann–Whitney U test.

**Table 3:** Age differences in clozapine dose and blood concentrations of clozapine, norclozapine, and clozapine N-oxide, as well as clozapine/norclozapine and clozapine/dose ratios.

Dose, Blood Levels, and Ratio	Age < 40			Age ≧ 40			*p*
	Mean	SD	Range	Mean	SD	Range	
Dose and concentrations before endpoint (*n* = 290/297)							
Dose (mg/day)	279	124	13–600	271	127	13–600	0.380
Clozapine (ng/mL)	461	297	43–1372	453	267	38–1330	0.976
Norclozapine (ng/mL)	344	234	38–1319	337	207	36–1016	0.901
Clozapine N-oxide (ng/mL)	92	51	12–253	88	50	10–390	0.254
Clozapine/norclozapine ratio	1.39	0.61	0.80–3.43	1.39	0.40	0.78–3.43	0.585
Clozapine/dose ratio	1.65	0.80	0.43–5.48	1.70	0.78	0.37–5.48	0.512
Dose and concentrations at end points (*n* = 83/84)							
Dose (mg/day)	326	129	75–600	322	117	125–600	0.829
Clozapine (ng/mL)	527	242	88–1119	524	280	106–1515	0.720
Norclozapine (ng/mL)	401	189	101–844	397	212	89–1131	0.665
Clozapine N-oxide (ng/mL)	110	50	35–234	107	54	33–295	0.659
Clozapine/norclozapine ratio	1.36	0.34	0.79–2.48	1.34	0.31	0.50–2.41	0.736
Clozapine/dose ratio	1.70	0.72	0.10–5.48	1.68	0.87	0.16–5.05	0.358

**Table 4:** Differences in blood parameters and cell counts between baseline and endpoint during clozapine treatment.

	Men					Women				
	Baseline		Endpoint			Baseline		Endpoint		
	Mean	SD (*n*)	Mean	SD (*n*)	*p*	Mean	SD (*n*)	Mean	SD (*n*)	*p*
Body weight (kg)	69.9	17.1 (89)	66.9	14.9 (89)	0.109	62.3	14.0 (62)	59.9	13.9 (62)	0.288
BMI (kg/m^2^)	24.2	5.1 (86)	23.2	4.4 (86)	0.161	25.6	6.4 (58)	24.2	5.3 (57)	0.252
Glucose (mg/dL)	95.5	17.9 (72)	103.3	22.5 (70)	0.018 *	94.9	20.8 (37)	104.1	28.1 (45)	0.023 *
HbA1c (%)	5.39	1.41 (74)	5.47	0.39 (72)	0.145	5.52	0.40 (37)	5.62	0.73 (44)	0.519
TC (mg/dL)	176	35 (63)	179	42 (62)	0.540	186	31 (36)	189	39 (39)	0.663
HDL (mg/dL)	51.5	12.5 (57)	56.5	11.2 (48)	0.025 *	57.3	16.4 (28)	59.3	15.8 (24)	0.440
TG (mg/dL)	123	78 (63)	136	79 (66)	0.224	96.0	38.0 (35)	107.1	41.8 (35)	0.254
WBC (/µL)	6540	2033 (75)	6800	2239 (97)	0.091	6080	2112 (39)	6615	1881 (65)	0.184
NEUT (%)	59.8	10.1 (75)	61.7	9.5 (96)	0.128	59.5	10.8 (38)	63.1	10.8 (64)	0.116
NEUT (/µL)	3938	1802 (75)	4302	2032 (95)	0.090	3794	1629 (37)	4271	1769 (64)	0.091
ALT (U/L)	29.3	17.8 (57)	33.6	20.3 (67)	0.227	23.6	14.4 (33)	29.3	17.4 (39)	0.075
AST (U/L)	23.7	10.5 (57)	26.9	16.7 (70)	0.980	21.4	11.3 (33)	23.6	10.9 (39)	0.116
γ-GTP (U/L)	34.4	26.1 (38)	28.5	20.0 (50)	0.330	21.1	10.7 (23)	24.0	20.0 (25)	0.917

Values are presented as mean ± SD. * indicates a significant difference (*p* < 0.05, Mann–Whitney U test).

## Data Availability

The data from this study are accumulated in the web-based input system, HCPMS (Hizen Clozaril Patients Monitoring System), which stores information on cases, including analysis results, dosage, background, blood test results, and clinical evaluations. Eleven participating institutions have access to this system; however, since it contains patients’ personal information, it cannot be made publicly available.

## Abbreviations

alanine aminotransferase (ALT); aspartate aminotransferase (AST); area under the receiver operating characteristic curve (AUC), body mass index (BMI), Brief Psychiatric Rating Scale (BPRS); confidence intervals (CI); dried blood spots (DBS); Diagnostic and Statistical Manual of Mental Disorders, Fifth edition (DSM-5); high-density lipoprotein (HDL); glucose, hemoglobin (HbA1c); high-performance liquid chromatography (HPLC); machine learning (ML); model-informed precision dosing (MIPD); neutrophil (NEUT); random forest (RF); receiver operating characteristic (ROC); SHapley Additive exPlanations (SHAP); standard deviation (SD); therapeutic drug monitoring (TDM); treatment-resistant schizophrenia (TRS); total cholesterol (TC); triglycerides (TG); white blood cell (WBC); volumetric absorptive paper discs (VAPDs); γ-glutamyl transpeptidase (γ-GTP).
